# CRISPR/Cas9-Mediated Knockout of *tnfaip1* in Zebrafish Plays a Role in Early Development

**DOI:** 10.3390/genes14051005

**Published:** 2023-04-28

**Authors:** Shulan Huang, Hongning Zhang, Wen Chen, Na Su, Changyue Yuan, Jian Zhang, Shuanglin Xiang, Xiang Hu

**Affiliations:** 1 State Key Laboratory of Developmental Biology of Freshwater Fish, College of Life Sciences, Hunan Normal University, Changsha 410081, China; huangshulan5920@163.com (S.H.); zhn@hunnu.edu.cn (H.Z.); chenwen@biochen.org (W.C.); 15074968356@163.com (N.S.); ycy010828@hunnu.edu.cn (C.Y.); zhangjian@hunnu.edu.cn (J.Z.); 2 Key Laboratory of Vascular Biology and Translational Medicine, Medical School, Hunan University of Chinese Medicine, Changsha 410208, China; 3 Engineering Research Center for Antibodies from Experimental Animals of Hunan Province, College of Life Sciences, Hunan Normal University, Changsha 410081, China

**Keywords:** *tnfaip1*, zebrafish, CRISPR/Cas9, embryonic development, RNA-seq

## Abstract

TNF α-induced protein 1 (*TNFAIP1*) was first identified in human umbilical vein endothelial cells and can be induced by tumor necrosis factor α (TNFα). Early studies have found that *TNFAIP1* is involved in the development of many tumors and is closely associated with the neurological disorder Alzheimer’s disease. However, little is known about the expression pattern of *TNFAIP1* under physiological conditions and its function during embryonic development. In this study, we used zebrafish as a model to illustrate the early developmental expression pattern of *tnfaip1* and its role in early development. First, we examined the expression pattern of *tnfaip1* during early zebrafish development using quantitative real-time PCR and whole mount in situ hybridization and found that *tnfaip1* was highly expressed in early embryonic development and, subsequently, expression became localized to anterior embryonic structures. To investigate the function of *tnfaip1* during early development, we constructed a model of a stably inherited *tnfaip1* mutant using the CRISPR/Cas9 system. *Tnfaip1* mutant embryos showed significant developmental delays as well as microcephaly and microphthalmia. At the same time, we found decreased expression of the neuronal marker genes *tuba1b*, *neurod1*, and *ccnd1* in *tnfaip1* mutants. Analysis of transcriptome sequencing data revealed altered expression of the embryonic development related genes *dhx40*, *hspa13*, *tnfrsf19*, *nppa*, *lrp2b*, *hspb9*, *clul1*, *zbtb47a*, *cryba1a*, and *adgrg4a* in the *tnfaip1* mutants. These findings suggest an important role for *tnfaip1* in the early development of zebrafish.

## 1. Introduction

TNF α-induced protein 1 (*TNFAIP1*) was first identified in human umbilical vein endothelial cells and can be induced by tumor necrosis factor α (TNFα) [1]. Human *TNFAIP1* is located on chromosome 17 and encodes a protein consisting of 316 amino acids. TNFAIP1 is a member of the PDIP1 subfamily of the KCTD protein family, which contains a conserved BTB/POZ domain at the N-terminal and functions through protein–protein interactions to form homologous or heterodimers [2]. The KCTD protein family can participate in the generation and transmission of cAMP signals, regulate neuronal function [3,4], and participate in the regulation of neuron development [5] and synaptic transmission [6]. At the same time, members of this protein family play an important regulatory role in the cardiovascular development of early embryos [7,8,9]. TNFAIP1 mediates DNA replication, repair, and cell cycle regulation by binding PCNA and a small subunit of polymerase δ (p50) [10]. Numerous studies have shown that TNFAIP1 is associated with proliferation [11], apoptosis [12,13,14], metastasis [15,16,17,18], and inflammatory responses [19] in a variety of tumors. In addition, TNFAIP1 is involved in chronic hepatitis B virus (HBV) infection, mediating immune tolerance to HBV [20]. Early studies found that TNFAIP1 is highly expressed at low pathogenic sites in *Caenorhabditis elegans* transgenic models of Alzheimer’s disease, and aberrant expression of the TNFAIP1 was detected in the brains of Alzheimer’s disease patients after death [21]. Amyloid-β (Aβ) induces the upregulation of TNFAIP1 expression, promoting neuronal cell damage [22,23,24]. The inhibition of TNFAIP1 expression inhibits nerve injury induced by oxygen–glucose deprivation/reperfusion [25]. TNFAIP1 and p-TNFAIP1 (phosphorylation of TNFAIP1-Ser280 site) are upregulated in the cerebral cortex and hippocampal neurons of APP/PS1 double transgenic mice, suggesting a close relationship between TNFAIP1 and Alzheimer’s disease pathogenesis [26]. TNFAIP1 expression is increased in the rat chronic constriction injury (CCI) model and influences the development of neuropathic pain in CCI rats [27]. TNFAIP1 has been found to interact with Rnd2 and Rnd3 to influence the dynamics of neuronal cell migration, neuronal branching, and dendritic spines in the embryonic cerebral cortex [28,29]. These studies reveal important functions of TNFAIP1 in nervous system development and Alzheimer’s disease. Thus far, studies on the function of *TNFAIP1* have mainly focused on pathological conditions such as tumors and Alzheimer’s disease, while there are few studies on the function of *TNFAIP1* under physiological conditions, in particular, the role of *TNFAIP1* in regulating embryonic development is still unknown. 

Species homology analysis revealed that the *TNFAIP1* gene is highly conserved across species, with 73% sequence homology between zebrafish and humans. Zebrafish are popular model organisms for development and disease research, and CRISPR/Cas9 gene editing of zebrafish is an important tool for functional genomics research [30]. Therefore, using the zebrafish model to study the role of *tnfaip1* in embryonic development may better contribute to understanding the function of *TNFAIP1* under physiological conditions.

In this study, we first detected the expression pattern of *tnfaip1* at different stages of zebrafish embryonic development and found that *tnfaip1* was highly expressed in early embryos, and, between 24 hpf and 72 hpf, the expression of *tnfaip1* was mainly localized in the anterior organs of the embryo. The *tnfaip1* mutant model was constructed using the CRISPR/Cas9 system. Phenotypic analysis revealed that *tnfaip1* mutant embryos showed significant developmental delay as well as microcephaly and microphthalmia. In addition, expression of neuronal marker genes was reduced in the mutant embryos. Analysis of transcriptome sequencing data revealed that the abnormal embryonic development was due to altered expression of genes associated with development in the mutant. Taken together, our results demonstrate the importance of *tnfaip1* in embryonic development, which provides a new theoretical basis for exploring the function of *tnfaip1* under physiological conditions.

## 2. Materials and Methods

### 2.1. Zebrafish Husbandry

Zebrafish (Danio rerio) (TU strain) were obtained from the China Zebrafish Resource Center (Wuhan, China). All wild-type and mutant zebrafish were bred under 14 h/10 h day/night cycle at 28.5 °C. Adult male and female zebrafish were mated in a 1:1 ratio to obtain embryos and staged in hours post-fertilization (hpf) or days post-fertilization (dpf). Fertilized eggs were cultured at 28.5 °C in E3 water (5 mM NaCl, 0.17 mM KCl, 0.33 mM CaCl_2_·H_2_O, 0.33 mM MgSO_4_·7H_2_O). Zebrafish experiments were performed according to the zebrafish book [31].

### 2.2. Whole Mount In Situ Hybridization

Zebrafish in situ hybridization experiments were performed according to the standard procedure for in situ hybridization reported previously [32]. The T7 promoter sequence was added to the 5’ end of the reverse primer used to synthesize the antisense RNA probe template for PCR amplification (Q5^®^ Hot Start High-Fidelity 2× Master Mix, M0494, NEB, Ipswich, MA, USA) with a cDNA template from 72 hpf wild-type zebrafish embryos. Digoxigenin-labelled antisense RNA probes were synthesized using T7 RNA polymerase (EP0111, Thermo, Waltham, MA, USA) and DIG RNA Labeling Mix (11277073910, Roche, Basel, Switzerland). After 24 hpf, the embryos were permeabilized with proteinase K: 24 hpf (2 min), 48 hpf (30 min), 72 hpf (45 min). DIG-labelled probes were detected using an alkaline-phosphatase-conjugated anti-DIG Fab fragments (11093274910, Roche, Switzerland), followed by staining with nitro blue tetrazolium (NBT; N6876, Sigma, Burlington, MA, USA) and 5-bromo-4-chloro-3-indolyl phosphate (BICP; B8503, Sigma, USA). All primer sequences used for the synthesis of antisense RNA probes are listed in Supplementary Table S1.

### 2.3. Generation of tnfaip1 Mutants

Generation of a *tnfaip1* zebrafish mutant model was performed using CRISPR/Cas9 gene editing technology [30,33]. The *tnfaip1* knockout target sites were designed using the CHOPCHOP online website (https://chopchop.rc.fas.harvard.edu/) (accessed on 20 July 2020), and the target sequences are listed in Supplementary Table S2. The sgRNA for the target sites was synthesized using an in vitro transcription kit (HiScribe™T7 Quick High Yield RNA Synthesis Kit, E2050S, NEB, USA) and, subsequently, purified using an RNA purification kit (RC201, Vazyme, Nanjing, China). TrueCut™ Cas9 protein v2 (A36498, Waltham, MA, USA) was purchased from Invitrogen. Subsequently, the ~1 nL of 150 ng/μL sgRNA and 0.5 μg/μL Cas9 protein complexes were microinjected into one-cell stage embryos. The homozygous mutant of *tnfaip1* was obtained by crossing heterozygotes. Primer sequences to identify the genotype of the mutant are listed in Supplementary Table S2.

### 2.4. Transcriptome Sequencing and Data Analysis

Sibling and *tnfaip1^−/−^* embryos were harvested at 72 hpf, with 4 replicates in each group, frozen in liquid nitrogen, and sent to Majorbio (Shanghai, China) for RNA extraction and transcriptome sequencing. Clean data of each sample obtained by RNA-seq reached more than 7.3 Gb. The clean reads of each sample were mapped to the zebrafish reference genome (GRCz11, http://asia.ensembl.org/Danio_rerio/Info/Index;) (accessed on 25 December 2022). STAR-2.7.1a was used to map reads to reference genome [34]. Quantification of gene expression was performed using RSEM [35]. Differential gene expression analysis was then performed using the package “DESeq2” [36]. DEGs between groups (*tnfaip1^−/−^* vs. sibling) were identified using the criteria *p*-adjust < 0.05 and|log2 (fold change)| ≥ 1. Volcano plots were drawn using the R package “ggplot2”. Gene Ontology (GO) and KEGG Pathway enrichment analyzes were performed with the “clusterProfiler” R package, and corrected *p*-values < 0.05 were considered significantly enriched.

### 2.5. Quantitative Real-Time PCR

Embryonic samples were collected from zebrafish at various developmental stages, and total RNA was extracted using RNAiso Plus (9108, Takara, Okinawa, Japan). cDNA was, subsequently, synthesized using GoScript™ reverse transcriptase (A5003, Promega, Madison, WI, USA). In this study, *18SrRNA* was used as an internal reference gene used for detecting the relative expression of genes, and all primer sequences are listed in Supplementary Table S3. Real-time PCR was performed on a QuantStudio™ 5 System Real-Time PCR Detection System (ABI, Foster City, CA, USA) using TB Green^®^ Premix ExTaq™ II (RR820A, Takara, Japan), and all samples were performed in triplicate. Relative expression levels of mRNA were calculated using 2^−ΔΔCT^ and expressed as mean ± SEM.

### 2.6. Protein Extraction and Western Blotting 

Zebrafish embryos at 72 hpf wild-type and *tnfaip1^−/−^* were collected and lysed in RIPA lysis buffer (BL504A, Biosharp, Hefei, China) with a protease inhibitor cocktail (M5293, Abmole, Houston, TX, USA). After protein separation with 12% polyacrylamide gels, they were transferred to PVDF membranes, incubated with antibodies, and detected. The zebrafish Tnfaip1 antibody (1:200) was customized at BOSTER (Wuhan, China). The TUBULIN antibody was purchased from Affinity Biosciences (1:5000, AF7010). The secondary antibodies goat anti-Rabbit IgG was purchased from Affinity Biosciences (1:10,000, S0001).

### 2.7. Embryo Imaging

Embryos before 72 hpf were manually stripped of their chorion, and all photographed embryos were anesthetized with 0.003% tricaine (E10521, Sigma, USA). Embryos were fixed in biconvex slides with 1% low melting agarose. Image acquisition was performed with a fluorescent stereomicroscope (M205 FA, Leica, Wetzlar, Germany). All photographed embryos were subjected to body length and eye size measurements using Image J.

### 2.8. Statistical Analysis

All experiments were performed with at least three independent biological replicates. Data were analyzed with unpaired two-tailed *t*-tests using GraphPad Prism 6. All data are presented as mean ± SEM. *p* < 0.05 was considered statistically significant, *p* < 0.01 was considered highly statistically significant, and *p* > 0.05 was considered not statistically significant.

## 3. Results

### 3.1. Spatio-Temporal Expression Pattern of tnfaip1 in Zebrafish during Early Development

**Figure 1:**
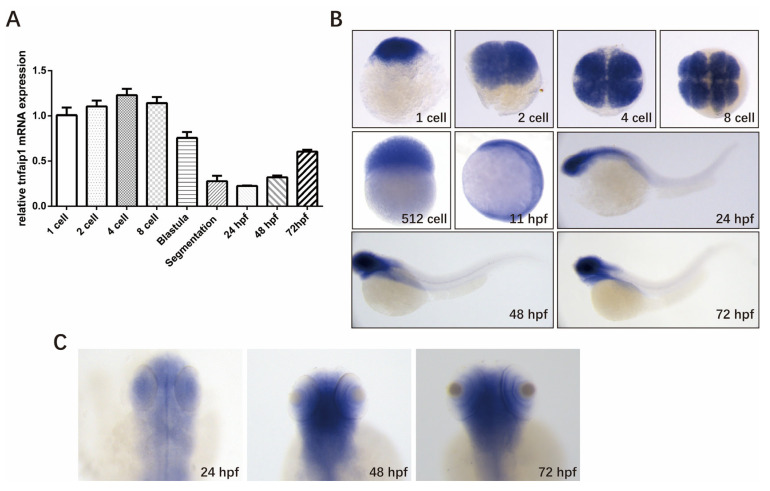
Spatio-temporal expression pattern of *tnfaip1* in zebrafish during early development. (**A**) qPCR detection of the relative expression level of *tnfaip1* mRNA during zebrafish embryo development, with *18SrRNA* as an internal control. (**B**) Whole mount in situ hybridization to detect *tnfaip1* expression during zebrafish embryo development. *n* = 15 per group; scale bar, 500 μm. (**C**) High magnification of *tnfaip1* expression in zebrafish 24, 48, and 72 hpf anterior organs. *n* = 15 per group; scale bar, 250 μm.

The expression pattern of a gene is the basis for its function. To investigate the role of *tnfaip1* during early zebrafish development, we first examined its expression during different developmental stages. Embryos were collected at different developmental stages, total RNA was extracted and reverse transcribed into cDNA, the qPCR primers specific for the *tnfaip1* gene were designed using the *18SrRNA* as an internal control, and quantitative real-time PCR was performed to detect the expression levels of *tnfaip1* mRNA at each stage of zebrafish embryo development. The results showed that *tnfaip1* was highly expressed during early zebrafish development, from 1 cell to 72 hpf (Figure 1A). In situ hybridization showed that *tnfaip1* was ubiquitously expressed during the cell period, and expression became localized to anterior embryonic structures between 11 hpf and 24 hpf (Figure 1B). *Tnfaip1* expression remained localized to the anterior embryo between 24 hpf and 72 hpf (Figure 1B,C). The in situ hybridization and qPCR results indicated that *tnfaip1* was a maternally expressed gene that functioned mainly through maternal expression in the early stage. *Tnfaip1* expression became localized to anterior embryo sometime between 11 hpf and 24 hpf and continued to be expressed in the anterior embryo after 24 hpf, revealing its possible involvement in the early development of multiple tissues and organs in the anterior structures of the zebrafish embryo.

### 3.2. Generation of the tnfaip1 Mutant in Zebrafish Using the CRISPR/Cas9 System

**Figure 2:**
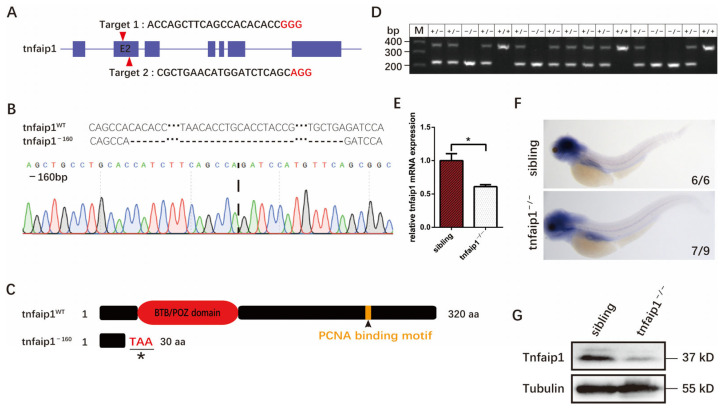
Generation of the *tnfaip1* mutant in zebrafish using the CRISPR/Cas9 system. (**A**) Gene structure of *tnfaip1* and location of CRISPR/Cas9 targets (red arrow), Target 1 and Target 2 are two knockout target sgRNAs, with the specific target sequence shown in black and the PAM (NGG) sequence in red. (**B**) The DNA sequencing peak and sequence of the *tnfaip1* mutant. DNA sequencing of the corresponding genomic region of zebrafish *tnfaip1* revealed a 160 bp deletion mutation. (**C**) The *tnfaip1^−160^* mutation resulted in a gene frame shift and early termination of the encoded protein. This mutation resulted in a protein truncated to 30 aa. The asterisks mark the termination codon. aa: amino acids. (**D**) Identification of genotype by DNA electrophoresis. +/+, wild type; +/−, heterozygote; −/−, homozygote. (**E**) Relative expression of *tnfaip1* mRNA at 72 hpf detected by qPCR and *18SrRNA* as an internal control. “*” represents *p* < 0.05, analyzed with unpaired two-tailed *t*-tests. (**F**) In situ hybridization to detect expression of *tnfaip1* in the mutants. (**G**) Decreased protein levels in *tnfaip1^−/−^* embryos at 72 hpf as measured by Western blot. Tubulin as an internal control.

To investigate the function of *tnfaip1* during early development, we generated a zebrafish *tnfaip1* mutant model using the CRISPR/Cas9 system. We designed two target sites on exon 2 of the *tnfaip1* in zebrafish and considered targeting efficiency, target location, and off-target effects (Figure 2A). The target site sgRNA and Cas9 protein were co-microinjected into 1-cell stage wild-type zebrafish embryos. Targeting efficiency was characterized after microinjection, and, after crossing the F0 and F1 generations, a mutant type with a 160 bp deletion in exon 2 was finally screened (Figure 2B and Figure S1). Sequence alignment analysis revealed that this mutation resulted in a shift in the open reading frame of *tnfaip1*, prematurely terminating the coding of the protein and resulting in a truncated protein (Figure 2C). To examine the effect of *tnfaip1* mutation on its mRNA and protein expression, we collected 72 hpf wild-type sibling and *tnfaip1* mutant embryos and performed qPCR, in situ hybridization, and Western blotting. The results of qPCR and in situ hybridization showed a decrease in mRNA levels by approximately 40% after mutation (Figure 2E,F). Western blotting showed decreased expression of Tnfaip1 protein in mutants (Figure 2G). However, a small amount of the protein was still present in the *tnfaip1* mutant, possibly due to residual maternal expression. These results indicated that we had successfully established a *tnfaip1* mutant model and can use this mutant for the next step in our research.

### 3.3. Mutation of tnfaip1 Affects the Early Development of Zebrafish Embryos

**Figure 3:**
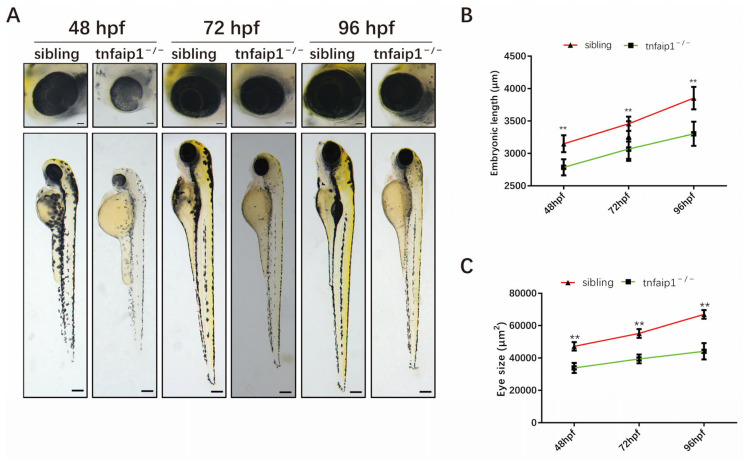
Mutation of *tnfaip1* in zebrafish affects early embryonic development. (**A**) Phenotypic analysis of 48–96 hpf wild-type sibling and *tnfaip1^−/−^* embryonic development. Scale bar: 750 μm. (**B**) Statistical analysis of 48–96 hpf wild-type sibling and *tnfaip1^−/−^* embryo body lengths. *n* = 30. “**” represents *p* < 0.01, analyzed with unpaired two-tailed *t*-tests. (**C**) Statistical analysis of 48–96 hpf wild-type sibling and *tnfaip1^−/−^* embryo eye sizes, *n* = 30. “**” represents *p* < 0.01, analyzed with unpaired two-tailed *t*-tests.

We crossed *tnfaip1* F1 heterozygous mutants to generate F2 embryos. Compared with wild-type sibling embryos at the same period, a small number of homozygous mutant embryos died during early development. This phenomenon may have been related to our early observation of pericardial edema and abnormal arrest of blood flow in homozygous mutant embryos. Subsequently, we observed the early development of these embryos under a stereomicroscope. We found that 48–96 hpf *tnfaip1* mutant embryos exhibited significant morphological abnormalities, including microphthalmia, smaller heads, and shortened body lengths (Figure 3A). Subsequently, we performed statistical analyses of body length (Figure 3B) as well as eye size (Figure 3C) of *tnfaip1^−/−^* embryos at different developmental stages.

**Figure 4:**
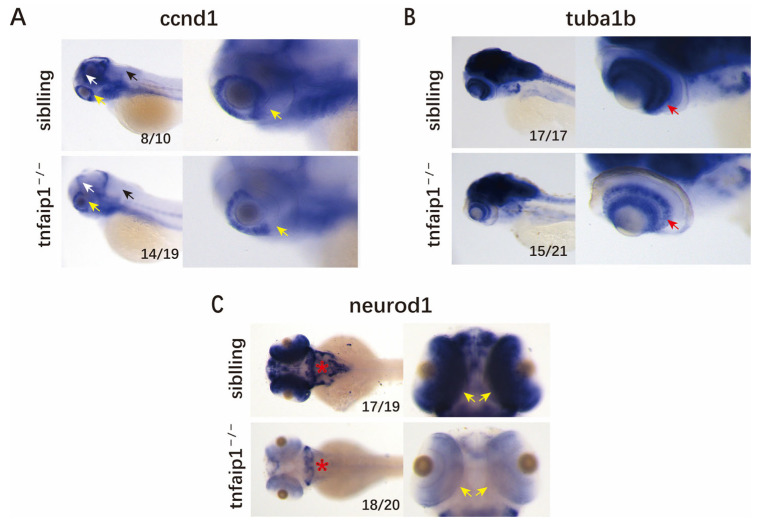
*Tnfaip1* mutation in zebrafish results in reduced expression of neuronal marker genes. (**A**) In situ hybridization assay for the 72 hpf wild-type sibling and *tnfaip1^−/−^* embryos neuronal marker gene *ccnd1*. Yellow arrows indicate the retinal; white arrows indicate the tectum proliferative zone; black arrows indicate the hindbrain. Scale bar: 250 μm. (**B**) In situ hybridization assay for the 72 hpf wild-type sibling and *tnfaip1^−/−^* embryos neuronal marker gene *tuba1b*. Red arrow indicates the retinal. Scale bar: 250 μm. (**C**) In situ hybridization assay for the 72 hpf wild-type sibling and *tnfaip1^−/−^* embryos neuronal marker gene *neurod1*. Red asterisks indicate the brain; yellow arrows indicate the retinal. Scale bar: 250 μm.

Early embryonic in situ hybridization showed that *tnfaip1* was predominantly expressed in the anterior structures, and we found that *tnfaip1* mutation resulted in microphthalmia and microcephaly. To identify the cause of this developmental malformation in *tnfaip1^−/−^* zebrafish, we collected 72 hpf wild-type sibling and *tnfaip1^−/−^* embryos for in situ hybridization assays for the neuronal marker genes *ccnd1*, *tuba1b*, and *neurod1*. The results showed that the neuronal marker genes *ccnd1* (Figure 4A), *tuba1b* (Figure 4B), and *neurod1* (Figure 4C) were significantly reduced in the mutants compared to the wild type. These results suggested that the reduction in *tnfaip1* expression affected the expression of neuronal marker genes, leading to microphthalmia and microcephaly.

### 3.4. Transcriptome Levels Are Altered in tnfaip1 Mutants

**Figure 5:**
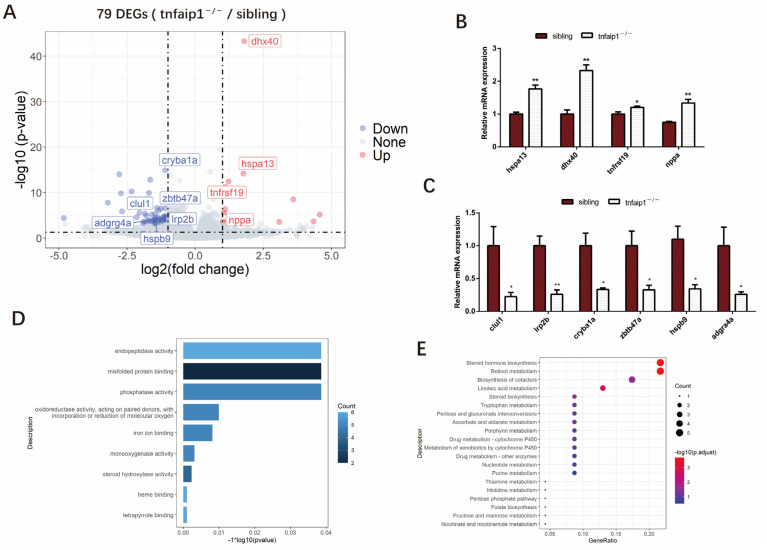
Identification of differentially expressed genes between siblings and *tnfaip1^−/−^*. (**A**) Volcano plot showing the overall differences in upregulated and downregulated genes in the mutants. Some differentially expressed genes are highlighted in the figure. DEGs, differentially expressed genes. (**B**,**C**) qPCR validation of differentially expressed genes associated with embryonic development in siblings and *tnfaip1^−/−^*. “*” represents *p* < 0.05, “**” represents *p* < 0.01, analyzed with unpaired two-tailed *t*-tests. (**D**) Gene Ontology (GO) enrichment analysis of differentially expressed genes from siblings and *tnfaip1* mutants. (**E**) KEGG pathway enrichment analysis of differentially expressed genes in siblings and *tnfaip1* mutants.

To explore the downstream molecular mechanisms by which reduction in *tnfaip1* affects the embryonic development of zebrafish, we collected 72 hpf embryos for transcriptome sequencing, with four independent biological replicates per group. Analysis of the sequencing results identified a total of 79 differentially expressed genes (DEGs), including 59 downregulated genes and 20 upregulated genes (Figure 5A). Altered expression of differentially expressed genes *dhx40*, *hspa13*, *tnfrsf19*, *nppa*, *lrp2b*, *hspb9*, *clul1*, *zbtb47a*, *cryba1a*, and *adgrg4a* in *tnfaip1* mutants was associated with early developmental regulation (Figure 5B,C). To determine the function of the differentially expressed genes of sibling and *tnfaip1^−/−^*, we performed GO functional and KEGG pathway enrichment analyses. The results showed that DEGs were mainly enriched in processes such as “misfolded protein binding”, “phosphatase activity”, and “oxidoreductase activity, acting on paired donors, with incorporation or reduction of molecular oxygen” (Figure 5D). KEGG pathway enrichment analysis showed that DEGs were mainly involved in the regulation of “steroid hormone biosynthesis”, “retinol metabolism”, “biosynthesis of cofactors”, and “nucleotide metabolism” (Figure 5E).

## 4. Discussion

*TNFAIP1* is thought to be associated with tumors and neurological disorders, and, to date, there have been no studies on the function of the *TNFAIP1* gene during early development. In the past, the study of the function of *TNFAIP1* have been limited to cellular models, and there is a lack of transgenic or knockout animal models of *TNFAIP1*, as zebrafish *tnfaip1* is highly homologous to the human *TNFAIP1* sequence. Therefore, this study focused on the expression pattern of *tnfaip1* in zebrafish during early development and the establishment of a zebrafish *tnfaip1* mutant model to lay the foundation for the elaboration of the biological functions of *tnfaip1* during early development.

The results of whole mount in situ hybridization and qPCR showed that zebrafish *tnfaip1* was a maternally expressed gene, and its expression level was relatively high in the early stage. With the depletion of maternal mRNA, its embryonic expression level began to increase gradually. Additionally, it was mainly expressed in the anterior structures of the embryo, including the head and eyes. This early developmental expression pattern of the zebrafish *tnfaip1* gene revealed its possible involvement in the early development and functional maintenance of multiple tissues and organs in the anterior structure of the embryo. In this study, for the first time, we constructed a zebrafish *tnfaip1* mutant model using CRISPR/Cas9 gene editing technology. Whole mount in situ hybridization and qPCR showed that the mRNA expression levels in the *tnfaip1* mutant were reduced by approximately 40%. We speculated that the reason for the incomplete suppression of mRNA expression in the mutant may have been due to incomplete nonsense-mediated mRNA degradation [37,38]. In addition, Western blotting showed that a small amount of the protein was still present in the *tnfaip1* mutant, possibly due to residual maternal expression. There was another possibility that the stop codon created by the mutation had some leakiness, which resulted in some expression of mRNA and protein remaining in the mutant. Due to our transcriptomic data showing no significant changes in the expression of the two orthologs of *tnfaip1*, *kctd10*, and *kctd13*, and no significant changes in the expression of other KCTD family paralogs were observed. 

In this study, we crossed *tnfaip1* F1 heterozygous mutants to generate F2 embryos. During early embryonic development from 48–96 hpf, we found that *tnfaip1* homozygous mutants exhibited markedly shortened body lengths, small eyes, and small heads. Due to the expression pattern of *tnfaip1* during early development and the observed microphthalmia and microcephaly in mutants, we hypothesized that *tnfaip1* may be required to maintain normal head and eye morphologies in zebrafish embryos. To further confirm this conjecture, we collected 72 hpf wild-type and *tnfaip1* mutant embryos for in situ hybridization of the neuronal marker genes *ccnd1*, *tuba1b*, and *neurod1*. The results showed that the expression of *ccnd1* in the tectum proliferative zone, hindbrain, and retina was significantly lower in the mutants than the wild-type sibling. The expression of *tuba1b* in retinal ganglion cells was significantly reduced in the mutants. It was significantly reduced expression of *neurod1* throughout the brain and retina of the mutants. These results suggested that microphthalmia and microcephaly caused by *tnfaip1* mutations may have been due to affecting the expression of neuronal marker genes during early development.

To explore possible downstream mechanisms by which *tnfaip1* mutations affect morphological abnormalities in early embryos, we collected 72 hpf wild-type sibling and *tnfaip1* mutant embryos for transcriptome sequencing. Analysis of the sequencing data revealed that most of the differentially expressed genes in the *tnfaip1* mutant were associated with early development. The altered expressions of *clul1*, *hspa13*, *dhx40*, *cryba1a*, *tnfrsf19*, *zbtb47a*, *lrp2b*, *hspb9*, and *adgrg4a* were closely associated with eye development, ocular diseases, formation of the nervous system, and embryonic development. *Clul1* was highly expressed in the retina, suggesting that it had an important role in the retina [39,40]. *Tnfaip1* mutation resulted in down-regulation of *clul1* expression, indicating that the microphthalmia caused by *tnfaip1* may have been due to the inhibition of *clul1* expression. *Hspa13* is a candidate gene for retinal degeneration, and its expression changes may lead to abnormal development of the retina [41]. GO functional enrichment analysis showed that differentially expressed genes were mainly involved in processes such as “misfolded protein binding”, “phosphatase activity”, and “oxidoreductase activity, acting on paired donors, with incorporation or reduction of molecular oxygen”; KEGG Pathway enrichment analysis results showed that the differentially expressed genes were mainly involved in the regulation of pathways such as “Steroid hormone biosynthesis”, “Retinol metabolism”, “Biosynthesis of cofactors”, and “Nucleotide metabolism”. These results suggested that *tnfaip1* mutations lead to alterations in the expression of a number of genes and associated signaling pathways that affected early embryonic development. However, studies on the function of *tnfaip1* in embryonic development are still in the initial stages. Our previous results showed that *tnfaip1* was highly expressed in multiple tissues and organs in the anterior structures of the embryo, and it was hypothesized that *tnfaip1* was likely to be involved in the formation and functional maintenance of these tissues and organs. Therefore, the early developmental regulation of specific tissues and organs by *tnfaip1* and details of its molecular mechanisms are worthy to further investigate in the future.

## 5. Conclusions

In this study, we found that *tnfaip1* was highly expressed during the early development of zebrafish. Between 11 and 24 hpf, the expression began to localize in the anterior organ of the embryo, and the expression of *tnfaip1* was still localized in the anterior embryo between 24 and 72 hpf. We generated a zebrafish *tnfaip1* mutant model using CRISPR/Cas9 gene editing technology. Additionally, using this mutant model, we observed morphological abnormalities in zebrafish embryonic development, including shortened body length, microphthalmia, and microcephaly. In addition, we found reduced expression of neuronal marker genes in *tnfaip1* mutants. RNA-seq data revealed altered expression of genes involved in the regulation of early development in *tnfaip1* mutants. Taken together, our findings suggest an important function of *tnfaip1* in the early development of zebrafish.

## Data Availability

All available raw data can be obtained from the corresponding author.

## Supplementary Materials

The following supporting information can be downloaded at: https://www.mdpi.com/article/10.3390/genes14051005/s1, Table S1: In situ hybridization primer sequences used in this study, Table S2: tnfaip1 sgRNA sequence and genotyping primer sequence used in this study, Table S3: qPCR primer sequences used in this study, Figure S1: Partial sequence of the *tnfaip1* mutant related to Figure 2.
