# Supplementary material for: CRISPR/Cas9-Mediated Knockout of tnfaip1 in Zebrafish Plays a Role in Early Development
Source: Genes (Basel). 2023 Apr 28;14(5):1005. doi: 10.3390/genes14051005 (PMC10217809; doi:10.3390/genes14051005)
Supplement: Supplementary file 1 [file genes-14-01005-s001.zip › genes-2302142-supplementary.pdf]

Supplementary Materials

Table S1. *In situ* hybridization primer sequences

Name	Sequence（5′-3′）
tnfaip1 probe F	TCTGCTGCACATCCATCGTT
tnfaip1 probe R	**TAATACGACTCACTATAGGG**TCACGCAAAGTTCAAGCAGC
neurod1 probe F	GGTGGGAATAGGCGTGAC
neurod1 probe R	**TAATACGACTCACTATAGGG**ACCCGAATAGTTTGAGCAG
tuba1b probe F	ATGCTGCTAATAACTATGCTCGTG
tuba1b probe R	**TAATACGACTCACTATAGGG**CCAACCTCCTCATAATCTTT
ccnd1 probe F	CAACTTCATCGCAAGCCCTC
ccnd1 probe R	**TAATACGACTCACTATAGGG**CGGTCATCAAAGCCACA

Table S2. *tnfaip1* sgRNA sequence and genotyping primer sequence

Name	Sequence（5′- 3′）
tnfaip1 sgRNA1	AATTAATACGACTCACTATAACCAGCTTCAGCCACACACCGTTTTAG AGCTAGAAATAGC
tnfaip1 sgRNA2	AATTAATACGACTCACTATACGCTGAACATGGATCTCAGCGTTTTAG AGCTAGAAATAGC
sgRNA scaffold	GATCCGCACCGACTCGGTGCCACTTTTTCAAGTTGATAACGGACTAG CCTTATTTTAACTTGCTATTTCTAGCTCTAAAAC
tnfaip1 genotyping F	GGGAAATCAGACCCATCGTGT
tnfaip1 genotyping R	AGGTTGCATCTTCTCTCAGGG

Table S3. qPCR primer sequences

Name	Sequence（5′-3′）
tnfaip1 qPCR F	GGTCTGATAGAACTGTTTATGCGTT
tnfaip1 qPCR R	GGCTGATCGTGATGCCAAAC
hspa13 qPCR F	ACTCAATAAACAGGGCGGCA
hspa13 qPCR R	CATATTGCTGCCGAACTCGC
dhx40 qPCR F	AGAAGTGGGCTACCAAGTGC
dhx40 qPCR R	AGCTGGGGTCTGCAAGAATC
nppa qPCR F	ATGGCCGGGGGACTAATTCT
nppa qPCR R	ATGCCTCTTCTGTTGCCAGG
tnfrsf19 qPCR F	GAGAGTGCTGGAGTTGTCCC
tnfrsf19 qPCR R	CAGCCTGCCATTGTCAGAGA
clul1 qPCR F	CTTTGGCAGACGTGTGTTGG
clul1 qPCR R	GACTCCCTGGCAGACTTCAC
lrp2b qPCR F	CGGATGGCTCACGCTATTCT
lrp2b qPCR R	GACGTAACTTGGGGCTCACA
cryba1a qPCR F	GCGCTTGCAGCTTTTTGTTG
cryba1a qPCR R	GCACGTTATCCATGCCACAC
zbtb47a qPCR F	CATGCGGACATGGCGAAAAA
zbtb47a qPCR R	CAGATCTGGCTGGAAGAGGC
hspb9 qPCR F	TGCAGAACCTGAGGAGTGAG
hspb9 qPCR R	GAGAAAAGCCTCGGGTGTCC
adgrg4a qPCR F	AGGACAGCGTACCCTGATTTAC
adgrg4a qPCR R	GCCAAATGCAGGATCGGATT

ATGTCAGGAGAGAGCTGCCTGCACCAGCTTCAGCCACACACCGGGCCCATC
CTGCCAGTCTCTTCAGTGGGCTACCCCAAGACTAACACCTGCACCTACCGTG
GTGTGACCGGAAATAAGTACGTTCAGCTCAATGTTGGTGGAAATCTGTACTA
CTCCACACTGCAGGTGCTCACTAGACAGGACACCCTGCTGAGATCCATGTTC
AGCGGCAAGATGGAGGTGCTCACAGATAAGGAAGGTTGGATCCTAATAGAT
CGCTGTGGGAAACACTTTGGCTCCATTCTCAGCT

Figure S1. Partial sequence of the *tnfaip1* mutant related to Figure 2. Orange bases are the start codon. Red bases are the stop codon. The green nucleotide sequence in the black dashed box are the deleted 160 bases.
